# Traditional medicine practitioners’ knowledge and views on treatment of pregnant women in three regions of Mali

**DOI:** 10.1186/1746-4269-9-67

**Published:** 2013-09-17

**Authors:** Hedvig Nordeng, Waled Al-Zayadi, Drissa Diallo, Ngolo Ballo, Berit Smestad Paulsen

**Affiliations:** 1 School of Pharmacy, University of Oslo, PO Box 1068, Blindern, Oslo N - 0316, Norway; 2 Division of Mental Health, National Institute of Public Health, Oslo, Norway; 3 Department of Traditional Medicine, National Institute of Research in Public Health, Bamako, Mali

**Keywords:** Traditional medicine, Pregnancy, Breast feeding, Mali, Traditional practitioner

## Abstract

**Background:**

Despite the widespread use of medicinal plants in Mali, knowledge about how traditional practitioners (TPs) treat pregnant and lactating women is lacking.

**Aim of the study:**

The aim of this study was to investigate how traditional practitioners in Mali treat common diseases and ailments during pregnancy.

**Methods:**

Data was collected through structured interviews of traditional practitioners in one urban (Bamako) and two rural areas (Siby and Dioila) in Mali. The TPs were interviewed about how they treat common diseases and ailments during pregnancy. They were also asked to name harmful plants in pregnancy and plants that could affect breast milk production. In addition, we asked about nine specific medicinal plants commonly used in Mali; *Opilia amentacea (syn. Opilia celtidifolia), Ximenia americana, Cola cordifolia, Combretum glutinosum, Parkia biglobosa, Trichilia emetica, Combretum micranthum, Lippia chevalieri* and *Vepris heterophylla.*

**Results:**

A total of 72 traditional practitioners (64% women, age: 34 to 90 years) were interviewed during an eight week period October 2011 to December 2011. They treated between 1 and 30 pregnant women with medicinal plants per months. We found a relatively high consensus for treatment of pregnant women with common diseases and ailments like nausea and dermatitis. The highest informer consensus was found for the treatment of malaria during pregnancy. TPs generally recommended pregnant women to avoid medicinal plants with bitter tastes like stem and root bark of *Khaya senegalensis* and *Opilia amentacea (syn. Opilia celtidifolia).* TPs distinguished between oral (potentially unsafe) and dermal use (safe) of *Opilia amentacea (syn. Opilia celtidifolia). Cola cordifolia* was used to facilitate labor.

**Conclusion:**

Experience and knowledge about treatment of pregnant women with medicinal plants was broad among the traditional practitioners in the three investigated regions in Mali. Collaborating with traditional practitioners on the safe use of medicinal plants in pregnancy may promote safer pregnancies and better health for mothers and their unborn infants in Mali.

## Background

Mali is a landlocked country in Western Africa south of Sahara with a population of approximately 14.5 million. About half the population lives below the international poverty threshold of US$1.25 a day. The country has the third highest birth rate in the world (45 births per 1000 inhabitants), and each woman gives birth to 6.4 children on average. Mali has the tenth highest maternal mortality rates in the world (830 deaths/100,000 live births), making pregnancy and childbirth one of the most dangerous periods during a woman’s life. Life expectancy for a woman is 55 years [1]. Major infections are food or waterborne diseases, and the vector borne disease malaria. Access to conventional medication and doctors is low (1 per 20 000 inhabitants).

In Mali, like many African countries 75% of the population depends on traditional medicine for primary health care [2,3]. Traditional medicine (TM) is according to the WHO, the total sum of knowledge, skills and practices based on the theories, beliefs and experiences indigenous to different cultures that are used to maintain health, as well as to prevent, diagnose, improve or treat physical and mental illnesses [2]. TM is sometimes also the only accessible and affordable source of health care - especially for poor patients. A study conducted by the WHO “Roll Back Malaria Program” in 1998 showed that in Mali more than 60% of children with high fever are treated at home with medicinal plants [4]. One of the key reasons cited for this was the easy accessibility of medicinal plants in rural areas.

Due to the global widespread use of traditional medicines, The WHO has developed a strategy to promote the safety, efficacy and quality of traditional medicine/complementary medicine (TM/CAM) in addition to adequate access and rational use of TM/CAM [2]. The strategy includes expanding the knowledge-base on TM/CAM and providing guidance on regulatory and quality assurance standards. Our project falls within the framework of this global strategy.

The traditional medicine practitioner, also called traditional healer, plays a pivotal role in the health care system in Mali. There is a well-established legal framework for practicing traditional medicine [5] and their services are highly sought and respected. Traditional practitioners (TPs) can obtain a certification after providing evidence of knowledge and experience. Knowledge is often handed down from generation to generation on how to prevent or treat diseases and ailments. There are 32 associations for practitioners of traditional medicine in the country and approximately one traditional medicine practitioner for every 500 inhabitants [2]. There is also a strong perception in the Malian culture that medicinal plants are effective.

Despite the key role of traditional practitioners in Mali, little is known about their views on treatment of pregnant women. This knowledge is important for several reasons. Firstly, we need to know how medicinal plants are being used to ensure that pregnant women are receiving treatment. For several diseases like malaria, going untreated during pregnancy is putting the mother and child at risk. Secondly, we need to identify potentially unsafe practices. If we know that a medicinal plant is unsafe, we can recommend women to avoid using them. Thirdly, we need to make sure that valuable information about medicinal plants in pregnancy is preserved.

We hypothesized that medicinal plants would be commonly used to treat pregnant women and that there would be a broad experience and knowledge about which medicinal plants were harmful for pregnant women. This study was undertaken to investigate the following specific research questions; 1) Which medicinal plants do TPs use for pregnant women?, 2) Which medicinal plants do TPs consider contraindicated in pregnancy?, 3) Which medicinal plants do they use for regulation of breast milk production? and 4) How is consensus between TPs when it comes to treatment of pregnant women with medicinal plants?

## Methods

**Figure 1:**
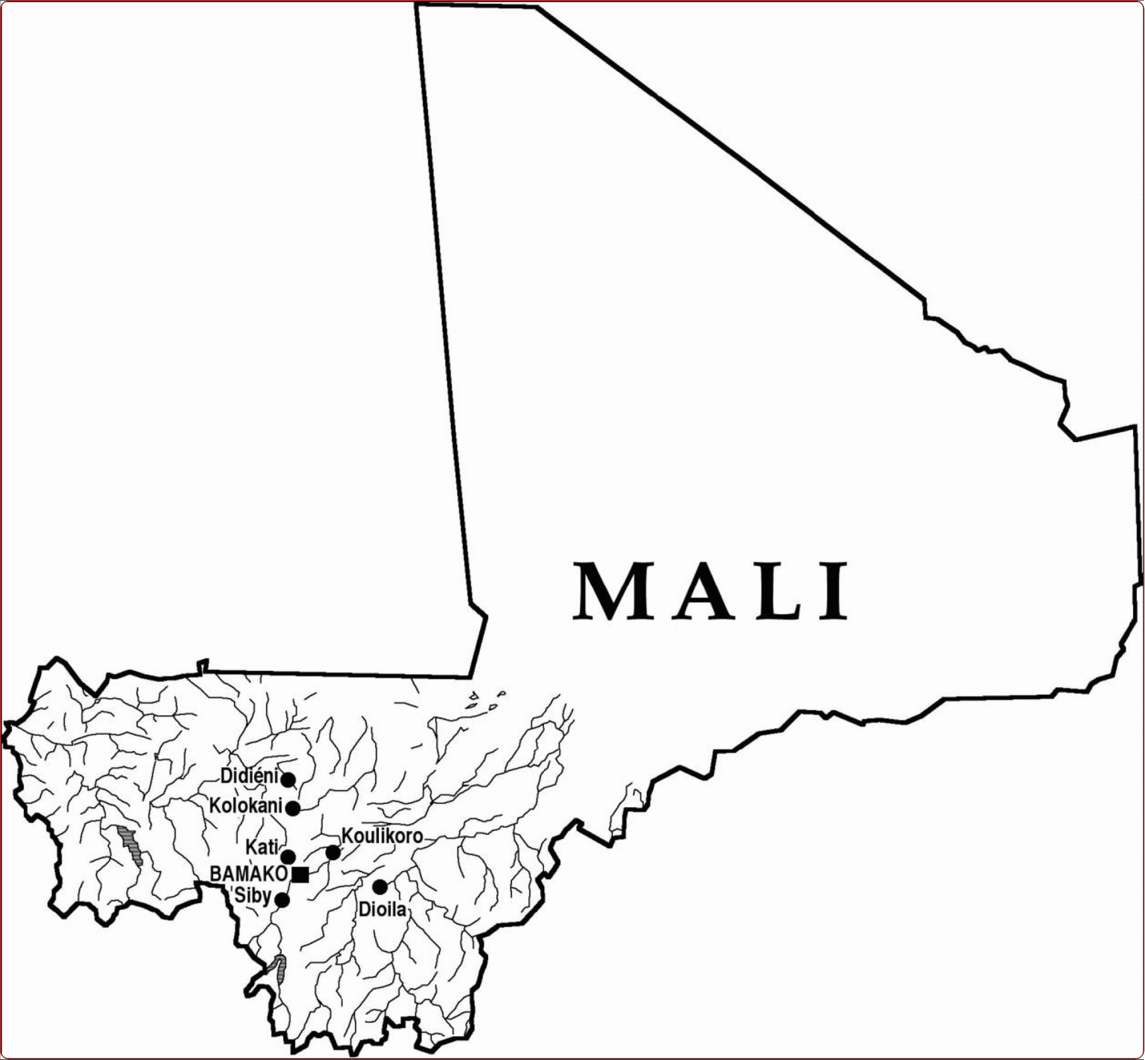
Map of Mali showing the three study locations; Bamako, Siby and Dioila.

The study is a descriptive interview-study of traditional practitioners in three regions in Mali; one urban region (region of Daoudabougou population size approximately 27 000, in Bamako – the capital of Mali) and two rural regions (Siby, 50 km south of Bamako, population size approximately 27 000 and Dioila, Commune of Banco, 130 km east of Bamako, population size approximately 29 000) (Figure 1). These regions were selected due to the geographic proximity to The Department of Traditional Medicine at the National Institute of Research in Public Health in Bamako and previous collaboration with the TPs in these regions [6-8]. The main ethnic groups in these three regions are the Bambara who are mostly farmers and speak the local language Bambara. Medicinal plants in these areas grow on Savanna plains or grasslands between equatorial forests and tropical deserts. Most of the TPs in the rural areas had a background as farmers whereas the TPs in the urban region were farmers or sometimes merchants. TPs interviewed were Bambara, Malinke.

All the TPs in the three districts were potentially eligible for study inclusion. Each TP in these districts had an equal chance of being selected (random sampling). TPs were approached in their homes and all present were invited for interviews. Prior to inclusion the TPs were explained the purpose of the study. All invited TPs accepted to participate in the study and consented on the uses of the data. They were informed that the data would be used for a scientific publication. The interviews were performed during the period October 2011 to December 2011 in Bambara, the local language, at the TPs homes with a plant taxonomist at the Department of Traditional Medicine at the National Institute of Research in Public Health, Mali, as interpreter. The interviews with the TPs were built on trust with the common goal of preserving and increasing the knowledge on medicinal plants and improving the health of the mother and child.

The interview was divided into four parts in the following order:

1) Treatment of thirteen diseases and common ailments during pregnancy, namely *nausea, tiredness, heartburn problems, skin problems, common cold, urinary tract infection, malaria, other infections (e.g. tuberculosis, worm infections), constipation, pain in back, neck or shoulder, headache and depression*,

2) Potentially harmful medicinal plants during pregnancy,

3) Medicinal plants that could impact on breast milk production. Plants that could either increase milk production or be harmful to breast feeding women were recorded.

4) Use in pregnancy of nine specific commonly used medicinal plants, namely *Opilia amentacea (syn. Opilia celtidifolia), Ximenia americana, Cola cordifolia, Combretum glutinosum, Parkia biglobosa, Trichilia emetica, Combretum micranthum, Lippia chevalieri and Vepris heterophylla.*

These nine plants were selected based on prior ethnopharmacological studies in Mali [6-8]. For each of these plants we calculated the degree of consensus between TPs using the fidelity level (FL) where FL = (Np/N)*100 [9]. Np is the number of TPs that claim use of a plant to treat a particular disease or ailment, and N is the total number of citations for any given disease or ailment. The discussion also included questions about which parts of the medicinal plants were used and how they were prepared. The traditional healers received a small monetary sum as compensation for lost work time (2000 CFA, approximately 4 US dollars = 3 Euro) and 20 cola nuts as is tradition in the Malian culture.

Voucher specimens of plants reported by TPs are located in the herbarium of the Department of Traditional Medicine at the National Institute of Research in Public Health in Bamako (Additional file 1).

The study was approved by the Regional Ethics Committee in Norway (REC Region South-East) and by the Norwegian Social Science Data Services (NSD). The study was conducted in accordance with international, national and institutional rules concerning the biodiversity rights. All computerized data were handled and stored anonymously. To ensure intellectual property rights a hand written list linking the TPs names to the questionnaires was kept separately and stored at the Department of Traditional Medicine, National Institute of Research in Public Health in Bamako.

## Results

A total of 72 traditional practitioners were interviewed during the eight weeks study period in Siby (31 healers), Dioila (31 healers) and Bamako (10 healers). The TPs had an average age of 67 years (range 34 to 90 years). In total, 46 healers were women (63.9%) and 26 men (36.1%). Most of them had extensive experience with treating pregnant women; in average they treated 13 pregnant women per month (range 1 to 30 women). Each interview lasted in average 41 minutes (range 8 to 97 minutes).

Table 1 presents an overview of the most commonly reported medicinal plants according to specific diseases and ailments during pregnancy. Over 40 different medicinal plants were cited for indications like nausea (50 plants), malaria (48 plants), tiredness (44 plants) and urinary tract infections (43 plants).

**Table 1:** Overview of medicinal plants cited by more than 7 TPs (≈10% of the TPs) according to indication in pregnancy

**Condition**	**Number of different**	**Most commonly used medicinal plants**	**Number of TPs**
	**medicinal plants cited**		**with citations**
Nausea	50	*Heliotropium indicum ¤:* drink boiled decoction of plant bundle	13
		*Combretum micranthum:* drink and/or wash body with boiled decoction of leaves	7
Tiredness	44	*Opilia amentacea (syn. Opilia celtidifolia):* shower or wash body with boiled decoction of leaves	11
		*Combretum micranthum:* drink small amount and/or wash body with boiled decoction of leaves	10
		*Afrormosia laxiflora ¤:* wash of boiled decoction of plant bundle	7
		*Guiera senegalensis ¤:* drink and/or wash body with boiled decoction of leaves	7
Heartburn	28	*Ximenia Americana:* drink boiled decoction of leaves Ingestion of a pulverized root/stem bark decoction is used to a lesser extent.	7
Skin problems	36	*Opilia amentacea (syn. Opilia celtidifolia):* shower or wash body with boiled decoction of leaves.	17
(Dermatitis)			
		*Trichilia emética:* wash body with boiled decoction of leaves	8
Common cold	32	*Guiera senegalensis ¤:* drink and/or wash body with boiled decoction of leaves	17
		*Lippia chevalieri:* drink tea of boiled decoction of plant bundle	10
Urinary tract infection	43	*Parkia biglobosa:* boiled decoction of stem bark as drink and/or wash of genitals	10
Fever of malaria	48	*Combretum micranthum:* boiled decoction of leaves often in combination with other medicinal plants	46
		*Trichilia emetica:* boiled decoction of leaves as wash or tea	32
		*Lippia chevalieri:* drink boiled decoction of plant bundle	26
		*Vepris heterophylla:* drink boiled decoction of leaves	21
		*Parkia biglobosa:* drink boiled decoction of stem bark or sometimes leaves	18
		*Combretum glutinosum:* drink boiled decoction of leaves	13
		*Opilia amentacea (syn. Opilia celtidifolia):* shower or wash body with boiled decoction of leaves	13
		*Sarcocephalus latifolius (syn. Nauclea latifolia) ¤:* boiled decoction of leaves, sometimes bark or roots	12
		*Mitragyna inermis ¤:* drink boiled decoction of leaves/plant bundle	10
Other infection	26	*Crossopteryx febrifuga ¤*#*:* drink decoction of fruit or leaves	7
Constipation	34	*Cassia alata ¤:* drink decoction of leaves	9
Pain in back, neck	35	-**	-
or shoulder			
Headache	29	*Daniella oliveri ¤:* boiled decoction of leaves for wash of head	12
		*Securidaca longipedunculata ¤:* pulverized root bark in water as inhalation, fumigation or wash of head	7
Depression	20	-**	-

**no medicinal plant mentioned more than twice. *¤* Medicinal plants cited that we did not specifically ask about. *#* Against tuberculosis.

Often the TP mentioned precautionary measures when treating pregnant women. This could be which plant parts not to use when pregnant (see below and Table 2), how to prepare the herbal medicine in a “milder” way or to only administer the herbal medicine dermally or as a fumigation instead of oral ingestion. For example, *Sarcocephalus latifolius (syn. Nauclea latifolia)* was recommended by several TPs against fever, but then as a “milder” water extract of leaves instead of trunk bark. Also, a boiled decoction of *Ximenia americana* was mentioned by 7 TPs to treat heartburn in pregnancy (contrary to grounded powder for oral ingestion). Some TPs stated that the boiled decoction of *Trichilia emetica* for fever should only be used externally as a wash when the woman was pregnant, whereas others considered use as a tea as acceptable in pregnancy.

Over 60% of the TPs recommended *Combretum micranthum* against fever of malaria. This plant was also used for other symptoms of malaria as tiredness (10 citations) and nausea (7 citations) often as a body wash of a boiled decoction of plant leaves.

Other infections mentioned were mostly tuberculosis (16 TPs) and worm infections (6 TPs). *Crossopteryx febrifuga* (7 citations) and *Sclerocarya birrea* (3 citations) were cited used against tuberculosis (fever) whereas everal medicinal plants were cited against worm infections (*Acanthospermum hispidum*, *Borreria stachydea, Cordia mixa, Hyptis spicigera, Ocimum basilicum, Opilia celtidifolia).*

Twenty medicinal plants were cited for use against depression, but no medicinal plant was mentioned more than twice. 76.4% of the TPs did not know of any medicinal plant for this condition. Thirty five plants were cited against pain in back, neck or shoulder, however none by more than 10% of the TPs. The most cited medicinal plant against this pain was *Guiera senegalensis* mentioned by six TPs (8.3%).

Table 2 presents the medicinal plants most commonly considered as harmful during pregnancy. Stem and roots of *Khaya senegalensis* and *Opilia amentacea (syn. Opilia celtidifolia)* were the plant parts which were mostly advised against during pregnancy. Because of their bitter taste, the traditional practitioners feared that they could induce abortion. Bitter taste is common for plants containing alkaloids, a type of plant compounds that often have pharmacologic properties [10,11]. For example, they emphasized that stem and roots of *Opilia amentacea (syn. Opilia celtidifolia)* could be harmful if ingested orally in large amounts, but could safely be used dermally (as a body wash). Leaves of *Opilia amentacea (syn. Opilia celtidifolia)* were not considered harmful. Likewise, a boiled decoction of *Ximenia americana* leaves was not considered strictly contraindicated in pregnancy.

**Table 2:** Overview of the top ten medicinal plants considered harmful by the traditional practitioners (TPs) and support in the literature for maternal-fetal use and toxicity

**Name of medicinal**	**No. TPs who**	**Plant parts**	**Relevant traditional use**	**Relevant toxicological and pharmacological data [**[10]**,**[12]**-**[14]**]**
**plant**	**considered the**			
	**plant harmful**			
*Khaya senegalensis*	14	Stem and root bark	Stem and root bark are used as arrow and fishing poison. The bark used as a purgative and as an emetic.	No contractile effect was found on the uterus of pregnant rat [15]. In high doses extracts of the plant was shown to be hepatotoxic [16].
*Opilia amentacea (syn. Opilia celtidifolia)*	11	Stem and roots	The stem and roots are used as diuretic and purgative remedies.	Reported variable effect on non-pregnant and pregnant uterus.
*Cassia sieberiana*	7	Root bark	The root bark has a use as a purgative and has a strong bitter taste. The plant is also used against infertility.	Hepatotoxic and neurotoxic effects were shown on rats [17]. Hepatotoxic effects was shown after long time use of extracts of *C. sieberiana*[18].
*Sarcocephalus latifolius (syn. Nauclea latifolia)*	5	Stem and root bark	Roots used as arrow poison. Root and stem bark are used as a purgative and to provoke vomiting.	The plant contains indol-alkaloids and saponins that often are toxic products, but they are relatively little soluble in water. It has been shown that root extracts will reduce the effect of oxytocin, ergometrine and acetylcholine induced uterus contractions that normally will stimulate birth [19].
*Trichilia emetica*	5	Bark	The bark is used as a purgative and as an emetic in small doses.	No toxicity data.
*Securidaca longipedunculata*	4	Roots	Roots used as arrow poison and to commit suicide. Also traditionally used against menstruation problems.	The root appears to be toxic in large doses, possibly due to a saponin. The roots also contain methyl salicylate that may cause harmful effects [20].
*Anogeissus leiocarpa*	3	Bark	A cold infusion is given to new born babies to drink. It is a laxative, and the plant is also used to treat diarrhea.	The bark has a high content of tannins, so are the leaves as well. No toxic effects reported in the literature.
*Adansonia digitata*	2	Bark	Infants are given extracts of the bark for gaining weight [21].	No toxic effects reported in the literature.
*Detarium microcarpum*	2	Root	The root is used against various ailments as vaginal discharge, sterility and fungal infections.	No toxic effects reported in the literature.
*Ximenia americana*	2	Leave, roots	Leaves and roots are traditionally used against dysmenorrhea, diarrhea, nausea, heartburn and jaundice.	Cyanogenic compounds are present which may cause harmful effects [22]. The bark has astringent properties due to the content of tannin.

*Ficus capensis* (20 citations) and *Euphorbia hirta* (8 citations) were most often recommended to increase breast milk production. Most commonly, the fruit of *Ficus capensis* was pulverized, dissolved in water and ingested orally together with bran*.* The fruit of *Ficus capensis* and milk juice of *Euphorbia hirta* have been used to stimulate lactation in several African countries [12]. The fruit of *Ficus capensis* can either be ingested orally or made into an ointment applied to the breast while the plant milk of *Euphorbia hirta* can be drunk, plant material chewed or massaged onto the woman’s breast. There is some evidence to support a positive impact of *Euphorbia hirta* on breast milk production [12].

Four TPs mentioned four different medicinal plants that should not be used by breast feeding women: *Cassia sieberiana, Khaya senegalensis, Opilia amentacea (syn. Opilia celtidifolia)* and *Securinega virosa.* Mostly, use of these plants was not recommended due to possible gastrointestinal adverse effects of the plants for the mother and breastfeeding infant (diarrhea and gastrointestinal pain). Four TPs also recommended breast feeding women with newborns to be careful about some foods as beans and honey.

Table 3 presents an overview of the nine medicinal plants we specifically asked about. These were also the most commonly reported medicinal plants in this study, in addition to *Guiera senegalensis* (cold; 17 citations, tiredness; 7 citations) and *Heliotropium indicum* (nausea, 13 citations). There was high consensus for the following medicinal plants *Combretum micranthum, Trichilia emetica, Vepris heterophylla, Lippia chevalieri* and *Parkia biglobosa* against malaria, *Opilia amentacea (syn. Opilia celtidifolia)* against dermatitis and malaria, *and Cola cordifolia* to facilitate labor.

**Table 3:** Overview of the nine most common medicinal plants in Mali according to indication of use and Fidelity level (FL) among the traditional practitioners

**Medicinal plant**	**Indication**	**Most common plant parts used**	FL (%)*
*Cola cordifolia*	Facilitate labor	Leaf, fruit, stem bark	29.6
	Tiredness		18.5
*Combretum glutinosum*	Malaria	Leaf	32.5
	Tiredness		12.5
*Combretum micranthum*	Malaria	Leaf	82.1
	Nausea		12.5
	Tiredness		17.9
*Lippia chevalieri*	Malaria	Leaf, root	41.9
	Cold		16.1
	Strengthen immune system		14.5
	Appetite stimulant		9.7
*Opilia amentacea (syn. Opilia celtidifolia)*	Dermatitis	Leaf	36.2
	Malaria		27.7
	Tiredness		23.4
	Stimulant of appetite		17.0
*Parkia biglobosa*	Malaria	Stem bark, leaf, fruit	36.7
	Urinary tract infection		20.4
	Internal wounds		12.2
*Trichilia emetica*	Malaria	Leaf, root	71.1
	Dermatitis		17.8
	Tiredness		11.1
	Chronic pain		11.1
*Vepris heterophylla*	Malaria	Leaf	65.6
	Constipation	Leaf (roots)	15.6
*Ximenia americana*	Heartburn		19.4
	Abdominal pain		13.9
	Prevent fetal disease		

Fidelity level (FL) = (Np/N) * 100 where Np is the number of TPs that claim use of a plant to treat a particular disease or ailment, and N is the total number of citations for any given disease or ailment [9].

*Opilia amentacea (syn. Opilia celtidifolia)* (N = 47), *Ximenia americana* (N = 36), *Cola cordifolia* (N = 27), *Combretum glutinosum* (N = 40), *Parkia biglobosa* (N = 49), *Trichilia emetica* (N = 45), *Combretum micranthum* (N = 56), *Lippia chevalieri* (N = 62) and *Vepris heterophylla* (N = 32).

## Discussion

To our knowledge this is the first study to investigate how traditional practitioners treat pregnant and lactating women. Using a plant taxonomist with knowledge of the local language and culture as interpreter was essential to capture essential information about various medicinal plants and establish confidence and trust with the traditional practitioners. It was clear that the traditional practitioners felt confident and had long experience with treating pregnant women and that they played an essential part of maternity care for pregnant women in these areas in Mali. Importantly, they recognized that some plants could have harmful effects for pregnant women, that the effect would vary according to which part of the plant was used, how they were prepared and their administration route, and adapted their recommendations to whether the woman was pregnant or not. Their knowledge was also supported by scientific literature for plants such as *Khaya senegalalensis, Opilia amentacea, and Cassia sieberiana* (Table 2).

For the most common diseases and ailments they had a large range of medicinal plants to choose from, like malaria where 48 plants were mentioned with fidelity levels of 82% and 71%, and 66% for *Combretum micranthum, Trichilia emetica* and *Vepris heterophylla*, respectively.

An important message for health workers is that medicinal plants rich in alkaloids and traditionally used as purgatives should not be used in pregnancy. Their mode of action warrants great caution even if there is limited human evidence of toxicity in pregnancy. E.g. roots of *Securidaca longipedunculata*, but also stem and root bark of *Khaya senegalensis, Opilia amentacea (syn. Opilia celtidifolia), Cassia sieberiana, Sarcocephalus latifolius (syn. Nauclea latifolia).* As shown in Table 2 for most of these plants, relevant toxicological and pharmacological evidence comes from traditional use and some animal studies. Our findings fit well with a previous study that showed that TPs in the district of Bamako, Mali, have broad knowledge about plant toxicity [13].

Treatment of malaria is an important public health priority in Mali as in many African countries [4,23]. In our study the TPs explained that they identified and treated the symptoms of malaria. In this way the TP may provide medicines complementary to conventional malaria medication. Ideally, conventional malaria medicines should be used as described in WHO guidelines [24]. However, when access to conventional drugs or diagnostics, costs or cultural factors make these guidelines difficult or impossible to follow, medicinal plants may be the only alternative. Of note, a few TPs informed us that they sometimes referred patients to the doctor, thus indication of a possible collaboration between TPs and doctors.

There is still a way to go to “roll back” malaria in Mali [23,25]. Our previous studies have shown that there is a strong belief that malaria may be caused by evil spirits [26] and reports show that large parts of the population do not receive conventional drugs against malaria [4,27]. Educating TPs and health care personnel jointly and including TPs actively in the national campaigns against malaria could be an important way forward to meet this major public health challenge.

Perhaps not surprisingly, depression was not readily recognized and although 20 different medicinal plants were mentioned, no plant was mentioned more than twice. On the other hand, they treated symptoms that could be related to depression like lack of appetite and tiredness. In many African countries having a mental illness is still a taboo and patients with mental illnesses are consequently stigmatized [28,29]. Our results support a public health initiative to increase awareness about mental illnesses among the public and health care personnel.

The training and system for registration of traditional practitioners that has been set in place and incorporated into the health care system in Mali might have helped to preserve knowledge inherited through generations and legitimate its use in the society where access to conventional medicines is limited. For this model to promote safe motherhood it is essential that such a system is in place and that the risks and benefits of medicinal plants are weighted in a similar manner as conventional medicines. Our concern is that although traditional use has generated important knowledge about the safety of several medicinal plants in pregnancy, systematic studies on the safety commonly used medicinal plants in pregnancy are lacking. As pregnant women cannot be included in randomized controlled trials of obvious ethical reasons, priorities for future research should be characterization of the components and biological activity of commonly used medicinal plants in Mali and incorporating medicinal plants in pharmacovigilance systems in African countries. Likewise, health workers should be educated and encouraged to report adverse pregnancy outcomes (e.g. spontaneous abortion, malformations) after use of medicinal plants. By doing so society would gain knowledge about these plants teratogenic potential and enable signal detection of harmful plants to the mother and unborn child, respectively. We believe that collaborating with traditional practitioners may be an important asset in such future research. In addition, a study on pregnant women’s use and attitudes towards medicinal plants should be undertaken to get the patients perspectives about treatment of ailments and diseases in pregnancy. Although several studies have been published on the use of medicinal plants in pregnancy in other African countries as the Ivory Coast [30], Nigeria [31,32], Zambia [33] and Tanzania [34], indicating a widespread use of medicinal plants in African countries, no such study has previously been conducted in Mali.

The Department of Traditional Medicine at the National Institute of Research in Public Health are currently undertaking several pharmacognostic and an epidemiological study relevant to these research areas.

There are some limitations to the study that should be acknowledged. Firstly, this study was conducted in three regions in Mali, and may not be representative of the entire country. The TPs who participated in the study may feel more confident and therefore more willing to discuss their practices with us than less experienced ones. By including practitioners with a large age range and number of pregnant women treated per month, both genders and traditional practitioners from three regions in Mali we hope to have overcome this potential bias. Furthermore, time could be a limiting factor; to explore the details of how medicinal plants were used, the interview lasted in average over 40 minutes, and sometimes the TPs would become impatient to finish the interview. Our results should be interpreted with the advantages and limitations of our study in mind.

## Conclusion

Use of medicinal plants to treat pregnant women was common among the traditional practitioners in the three investigated regions in Mali. Experience and knowledge about a wide range of medicinal plants was found. To promote healthy pregnancies, more research needs to be conducted on the efficacy and safety of commonly used medicinal plants in pregnancy in Africa. Collaborating with traditional practitioners may be an important asset in both future research and public health priorities as improving maternal health in pregnancy and after childbirth.

## Competing interests

The authors declare that they have no competing interests.

## Authors’ contribution

HN, WA-Z, BSP and DD conceived of the study. NB and WA-Z conducted the interviews with the traditional healers and identified all medicinal plants described. WA-Z coded all the data. WA-Z and HN performed the statistical analysis. HN wrote the first draft of the manuscript. All authors contributed to interpretation of the results and contributed to the final manuscript. All authors read and approved the final manuscript.

## Authors’ information

HN Dr.Philos., MSc.Pharm. is a professor at the School of Pharmacy, University of Oslo, Norway and researcher at the Division of Mental Health, Norwegian Institute of Public Health, Oslo, Norway. The focus of her research is medication use and safety during pregnancy and breastfeeding.

WA-Z, MSc. Pharm. is a pharmacist. The work was his master thesis project at the School of Pharmacy, University of Oslo, Norway.

NB is a plant taxonomist at the Department of Traditional Medicine at the Faculté de Pharmacie, Université des Sciences, des Techniques et des Technologies de Bamako and the National Institute of Research in Public Health, Bamako, Mali.

DD Ph.D. MSc.Pharm. is a professor and head of Department of Traditional Medicine at the Faculté de Pharmacie, Université des Sciences, des Techniques et des Technologies de Bamako and the National Institute of Research in Public Health, Bamako, Mali.

BSP PhD, MSc.Pharm. is a professor at the School of Pharmacy, University of Oslo, Norway. The focus of her research is natural products chemistry with focus on bioactive polysaccharides and ethnopharmacology.

## Supplementary Material

Additional file 1 Voucher specimens of medicinal plants located in the herbarium of the Department of Traditional Medicine at National Institute of Research in Public Health, Mali. Click here for file
