# Supplementary material for: Traditional medicine practitioners’ knowledge and views on treatment of pregnant women in three regions of Mali
Source: J Ethnobiol Ethnomed. 2013 Sep 17;9:67. doi: 10.1186/1746-4269-9-67 (PMC3851135; doi:10.1186/1746-4269-9-67)
Supplement: Additional file 1 — Voucher specimens of medicinal plants located in the herbarium of the Department of Traditional Medicine at National Institute of Research in Public Health, Mali. [file 1746-4269-9-67-S1.doc]

**Additional file 1.** Voucher specimens of medicinal plants located in the herbarium of the Department of Traditional Medicine at National Institute of Research in Public Health, Mali

| *Plant name* | Local name | *Voucher specimen number* |
| --- | --- | --- |
| *Adansonia digitata* | Sira | 0133/DMT |
| Afrormosia laxiflora | Kolo-kolo | 1081/DMT |
| *Anogeissus leiocarpa* | Ngalama | 0376/DMT |
| Cassia alata | Ko-n’taba | 2487/DMT |
| *Cassia sieberiana* | Sindia | 0971/DMT |
| *Cola cordifolia* | N’tabanoko/Ntaba | 1331/DMT |
| *Combretum glutinosum* | Cangara | 0533/DMT |
| *Combretum micranthum* | Ngolobe | 0587/DMT |
| *Crossopteryx febrifuga* | Balembo | 0157/DMT |
| Daniella oliveri | Sana | 0190/DMT |
| *Detarium microcarpum* | Ntabacoumba | 0017/DMT |
| *Euphorbia hirta* | Demba sindji | 0992/DMT |
| *Ficus capensis* | Toro | 2288/DMT |
| *Guiera senegalensis* | Kudje | 0749/DMT |
| *Heliotropium indicum* | Nonsikou | 0759/DMT |
| *Khaya senegalensis* | Diala | 0731/DMT |
| *Lippia chevallieri* | N’ganiba | 0001/DMT |
| *Mitragyna inermis* | Djun | 2263/DMT |
| *Opilia amentacea*  *(syn. Opilia celtidifolia)* | Korôgé | 0904/DMT |
| *Parkia biglobsa* | Nere | 0285/DMT |
| *Sarcocephalus latifolius*  *(syn. Nauclea latifolia)* | Bari | 1117/DMT |
| *Securidaca longipedunculata* | Djoro | 0058/DMT |
| *Trichillia emetic* | Sula finzan | 0561/DMT |
| *Vepris heterophylla/Teclea sudanica* | Kita kanganiba | 2444/DMT |
| *Ximenia americana* | Ntonke/Hongbé | 0764/DMT |
